# Supplementary figures and images for: Anglers as potential vectors of aquatic invasive species: Linking inland water bodies in the Great Lakes region of the US
Source: PLoS One. 2023 Jul 20;18(7):e0276028. doi: 10.1371/journal.pone.0276028 (PMC10358920; doi:10.1371/journal.pone.0276028)

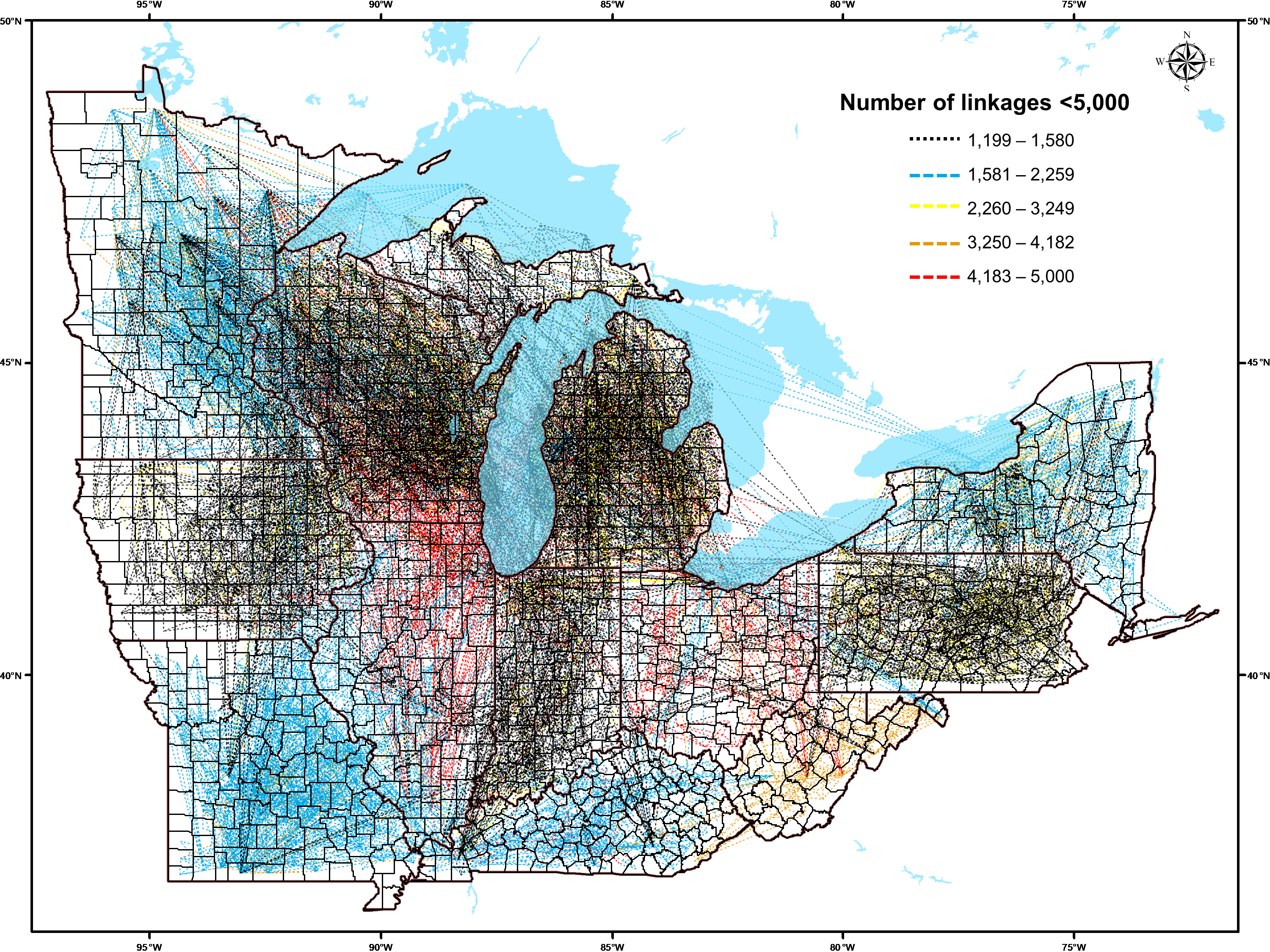

Supplement: S1 Fig — Connections and estimated magnitudes of movements of anglers among counties in the 13 US states surrounding the Great Lakes and upper Mississippi River basins. The estimated 14,766 links among counties in a single year demonstrate the likelihood of spread by anglers and the potential pathways of transfer of aquatic invasive species. Black dotted lines represent annual connections between counties numbering from 1199–1580; blue dashed lines range from 1581–2259; yellow dashed lines range from 2260–3249; orange dashed lines range from 3250–4182; and red dashed lines range from 4183–5000 angler trips between counties in a single year. (TIF) [file pone.0276028.s001.TIF]

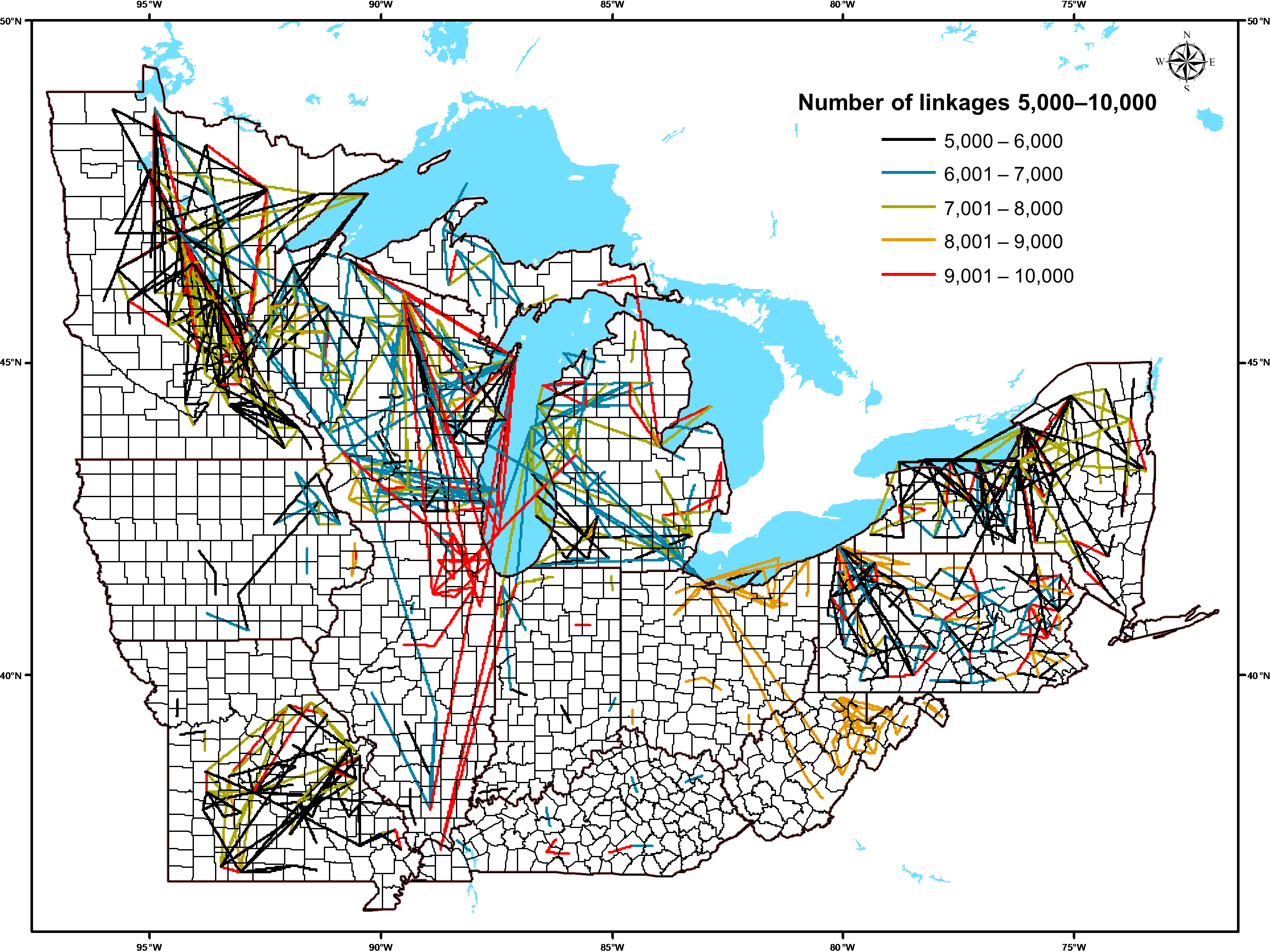

Supplement: S2 Fig — Connections and estimated magnitudes of movements of anglers among counties in the 13 US states surrounding the Great Lakes and upper Mississippi River basins. The estimated 14,766 links among counties in a single year demonstrate the likelihood of spread by anglers and the potential pathways of transfer of aquatic invasive species. Black lines represent annual connections between counties numbering from 5000–6000; blue lines range from 6001–7000; green lines range from 7001–8000; yellow lines range from 8001–9000; orange lines range from 8001–9000; and red lines range from 9001–10,000 angler trips between counties in a single year. (TIF) [file pone.0276028.s002.TIF]

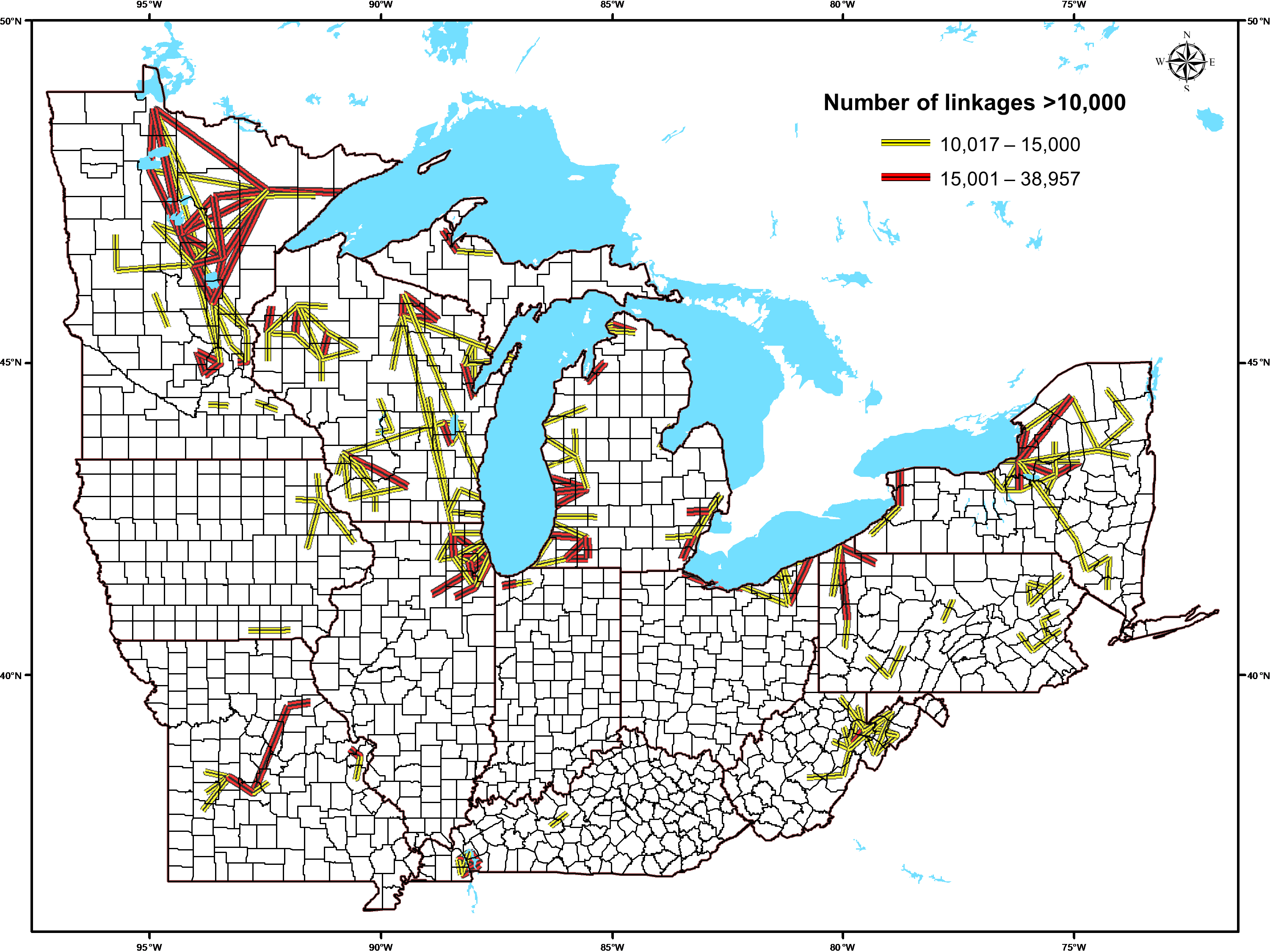

Supplement: S3 Fig — Connections and estimated magnitudes of movements of anglers among counties in the 13 US states surrounding the Great Lakes and upper Mississippi River basins. The estimated 14,766 links among counties in a single year demonstrate the likelihood of spread by anglers and the potential pathways of transfer of aquatic invasive species. Double-yellow lines represent annual connections between counties numbering from 10,017–15,000, and double-red lines range from 9001–10000 angler trips between counties in a single year. (TIF) [file pone.0276028.s003.TIF]

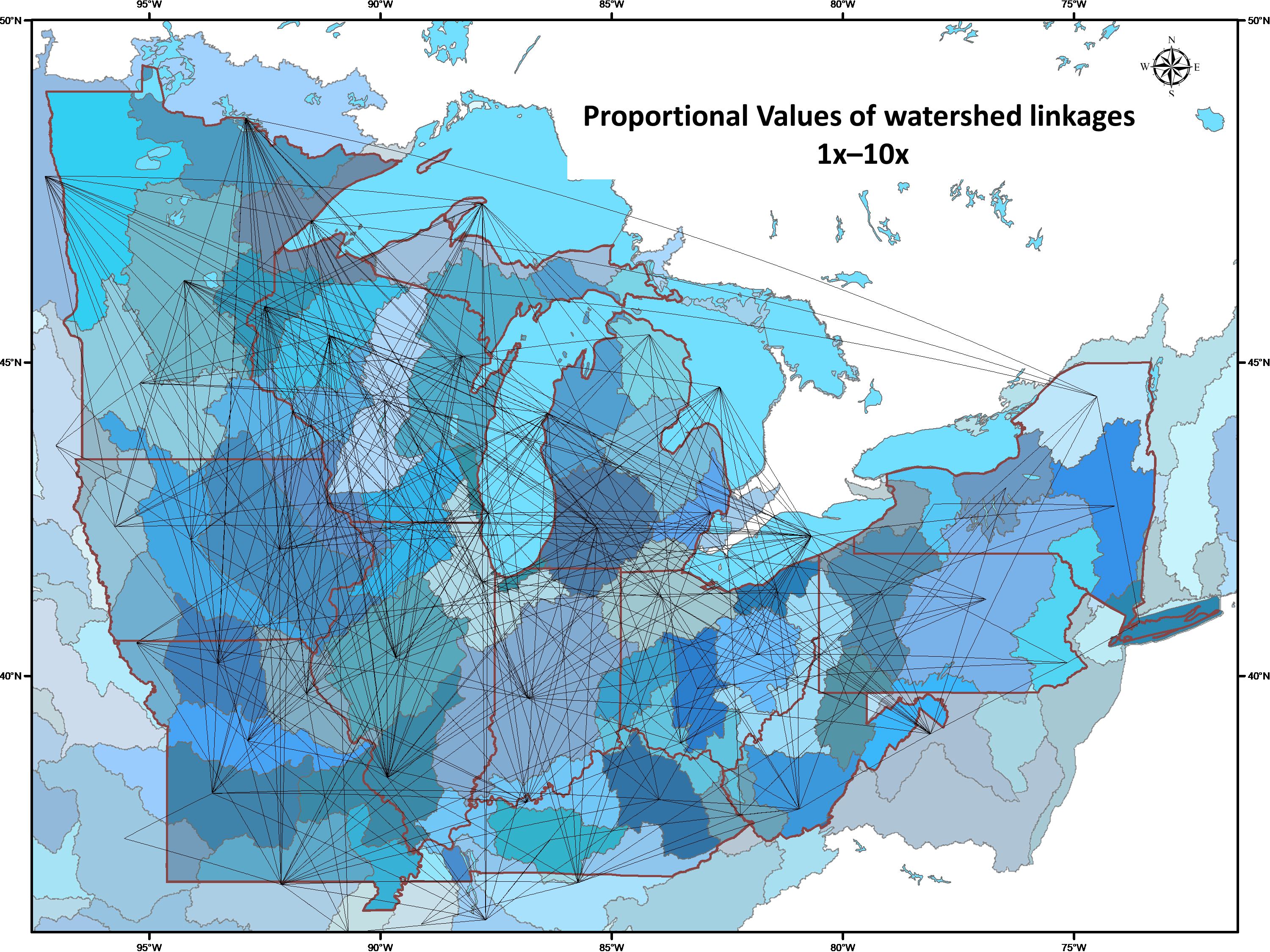

Supplement: S4 Fig — Lines showing the network of linkages among the 66 watersheds (HUC4 Hydrologic Units) that comprise the 12 US states surrounding the Great Lakes and upper Mississippi River basins. Lines between watersheds represent direct connections made by individual anglers fishing in multiple watersheds in the survey year. The multiple direct connections among watersheds demonstrate the potential for introduction and inter-basin spread of aquatic invasive species by anglers along linked pathways. Connecting lines represent the unweighted numbers of anglers among the 2576 survey participants who connected separate watersheds in a single year. Unweighted values, ranging from 1–10, represent proportions of the millions of licensed anglers in the study area. (TIF) [file pone.0276028.s004.TIF]

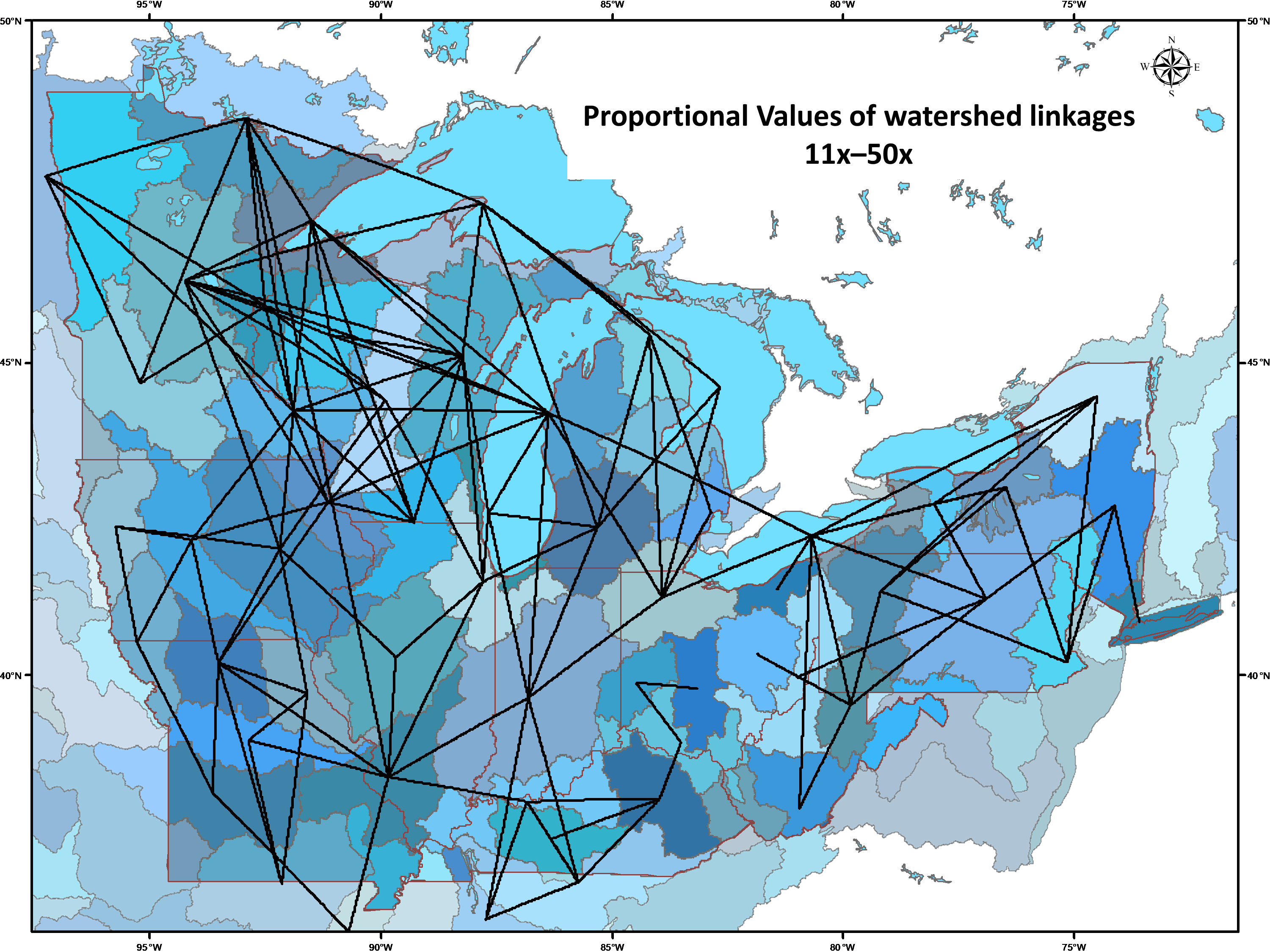

Supplement: S5 Fig — Lines showing the network of linkages among the 66 watersheds (HUC4 Hydrologic Units) that comprise the 12 US states surrounding the Great Lakes and upper Mississippi River basins. Lines between watersheds represent direct connections made by individual anglers fishing in multiple watersheds in the survey year. The multiple direct connections among watersheds demonstrate the potential for introduction and inter-basin spread of aquatic invasive species by anglers along linked pathways. Connecting lines represent the unweighted numbers of anglers among the 2576 survey participants who connected separate watersheds in a single year. Unweighted values, ranging from 11–50, represent proportions of the millions of licensed anglers in the study area. (TIF) [file pone.0276028.s005.TIF]

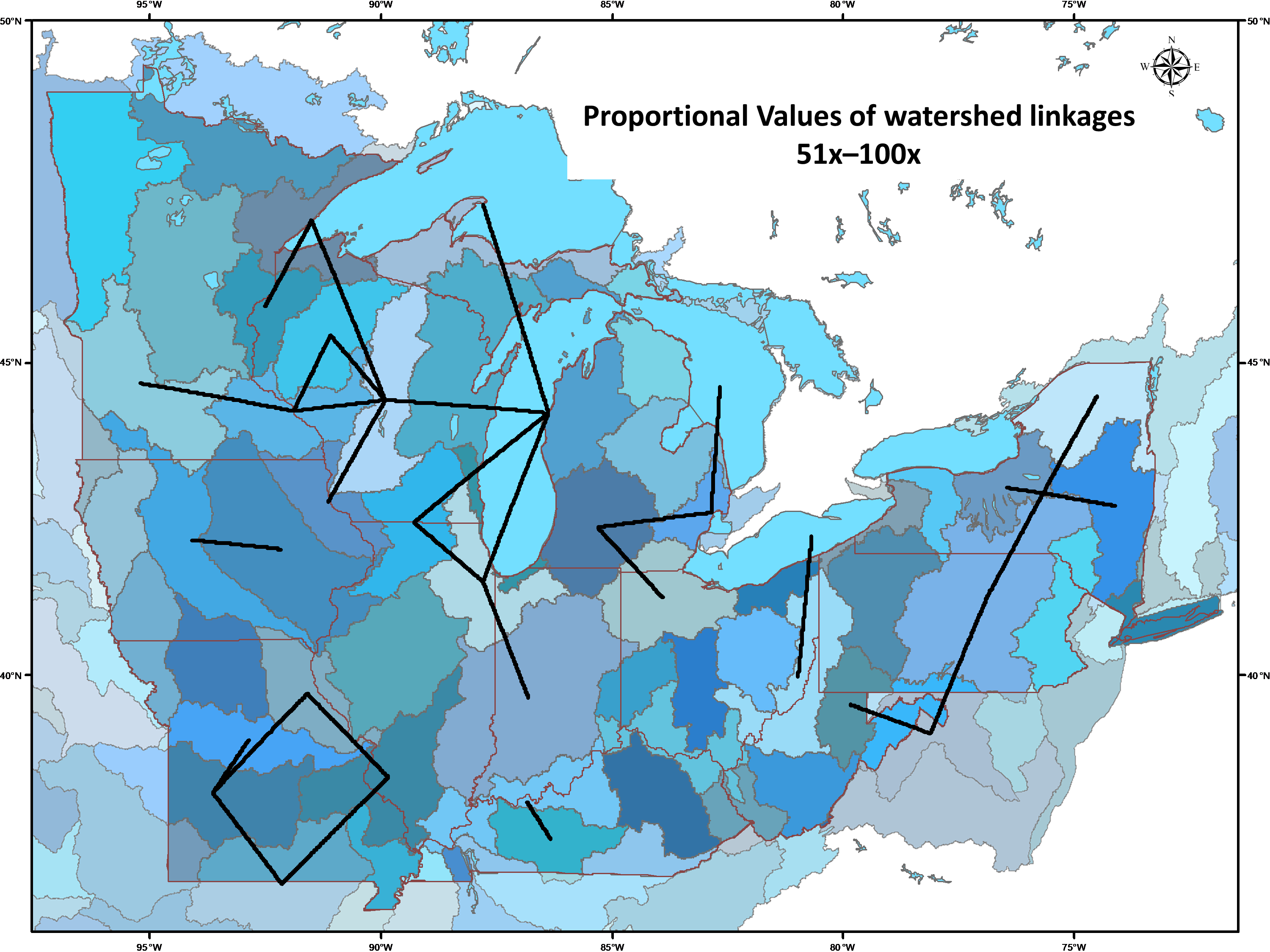

Supplement: S6 Fig — Lines showing the network of linkages among the 66 watersheds (HUC4 Hydrologic Units) that comprise the 12 US states surrounding the Great Lakes and upper Mississippi River basins. Lines between watersheds represent direct connections made by individual anglers fishing in multiple watersheds in the survey year. The multiple direct connections among watersheds demonstrate the potential for introduction and inter-basin spread of aquatic invasive species by anglers along linked pathways. Connecting lines represent the unweighted numbers of anglers among the 2576 survey participants who connected separate watersheds in a single year. Unweighted values, ranging from 51–100, represent proportions of the millions of licensed anglers in the study area. (TIF) [file pone.0276028.s006.TIF]

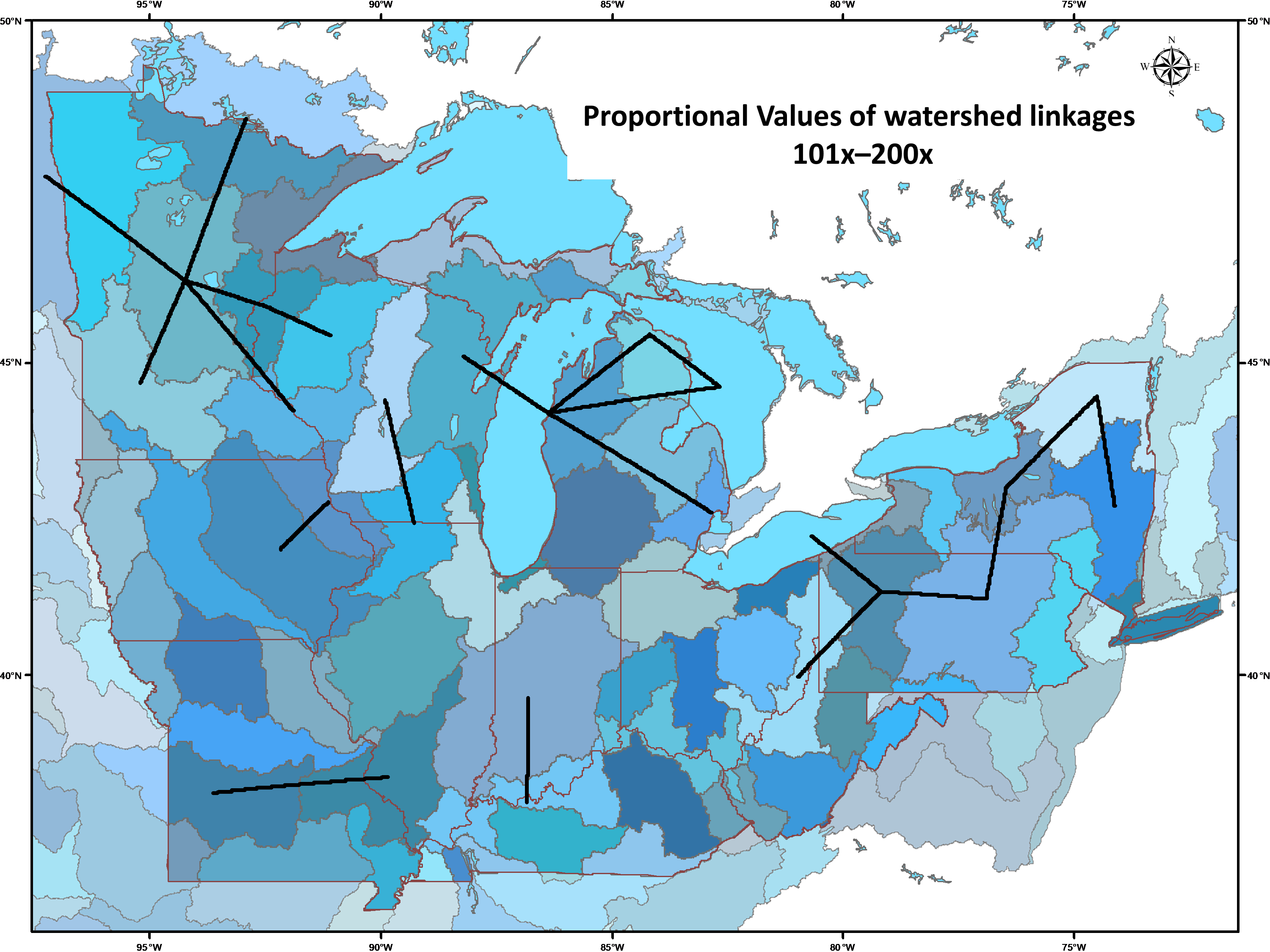

Supplement: S7 Fig — Lines showing the network of linkages among the 66 watersheds (HUC4 Hydrologic Units) that comprise the 12 US states surrounding the Great Lakes and upper Mississippi River basins. Lines between watersheds represent direct connections made by individual anglers fishing in multiple watersheds in the survey year. The multiple direct connections among watersheds demonstrate the potential for introduction and inter-basin spread of aquatic invasive species by anglers along linked pathways. Connecting lines represent the unweighted numbers of anglers among the 2576 survey participants who connected separate watersheds in a single year. Unweighted values, ranging from 101–200, represent proportions of the millions of licensed anglers in the study area. (TIF) [file pone.0276028.s007.TIF]

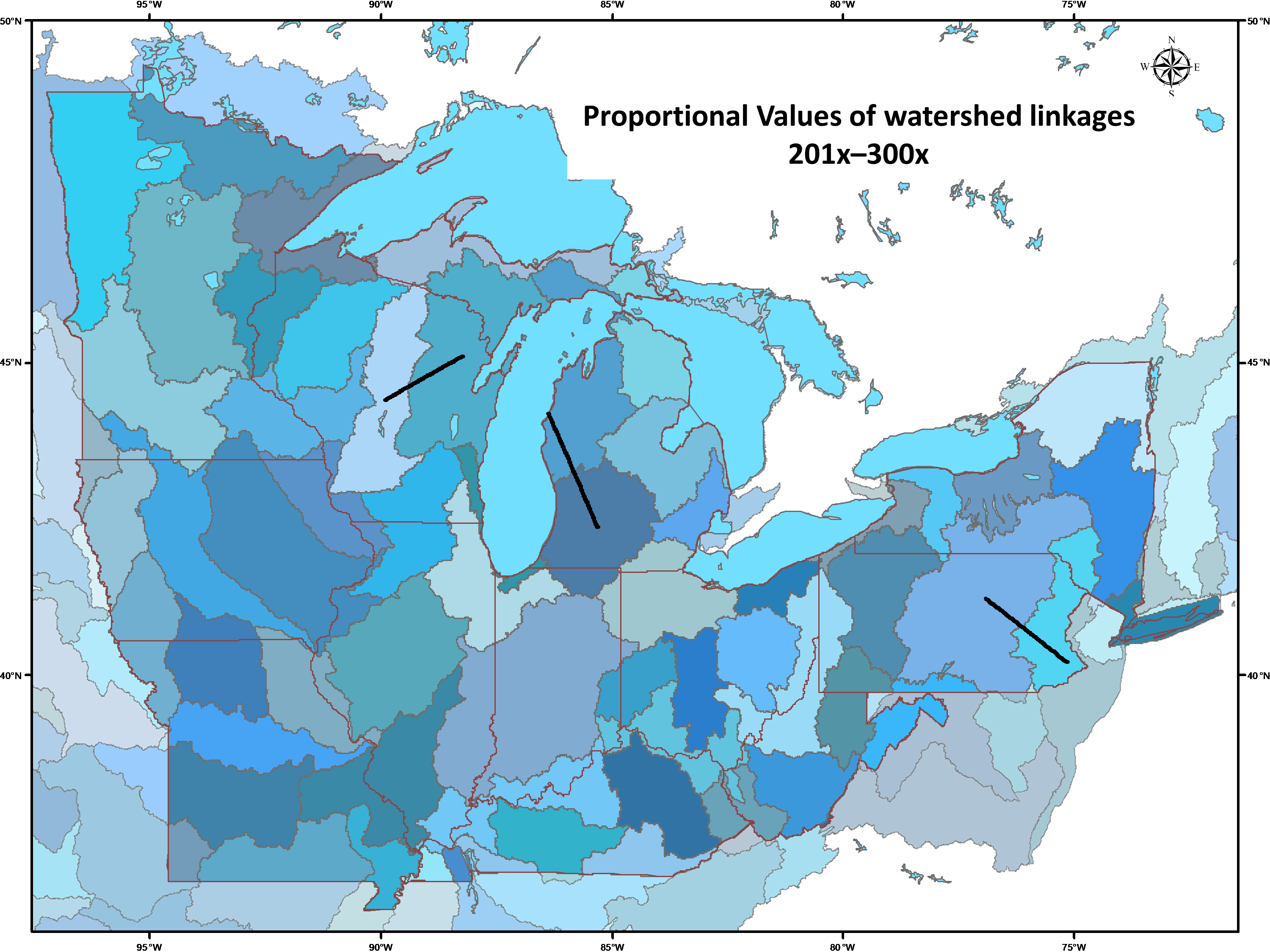

Supplement: S8 Fig — Lines showing the network of linkages among the 66 watersheds (HUC4 Hydrologic Units) that comprise the 12 US states surrounding the Great Lakes and upper Mississippi River basins. Lines between watersheds represent direct connections made by individual anglers fishing in multiple watersheds in the survey year. The multiple direct connections among watersheds demonstrate the potential for introduction and inter-basin spread of aquatic invasive species by anglers along linked pathways. Connecting lines represent the unweighted numbers of anglers among the 2576 survey participants who connected separate watersheds in a single year. Unweighted values, ranging from 201–300, represent proportions of the millions of licensed anglers in the study area; these three major linkages all connect watersheds to large metropolitan centers. (TIF) [file pone.0276028.s008.TIF]
